# Preparation of Lignin Carbon/Zinc Oxide Electrode Material and Its Application in Supercapacitors

**DOI:** 10.3390/molecules26123554

**Published:** 2021-06-10

**Authors:** Gaijuan Guo, Zijing Zhou, Jinda Li, Hong Yan, Fen Li

**Affiliations:** 1 School of Materials Science and Engineering, Harbin University of Science and Technology, Harbin 150040, China; guogaijuan616534@aliyun.com (G.G.); zzj15046043588@aliyun.com (Z.Z.); jinda_li@aliyun.com (J.L.); 2 Key Laboratory of Green Chemical Technology of College of Heilongjiang Province, Harbin 150040, China

**Keywords:** lignin carbon, ZnO, composite material, supercapacitors

## Abstract

In this paper, carbon/zinc oxide (LC/ZnO) composites were successfully synthesized and characterized by X-ray powder diffraction, field emission scanning electron microscopy, Fourier transform infrared spectroscopy, Raman, thermogravimetry, and N_2_ adsorption–desorption, and tested by electrochemical performance. Studies have shown that the morphology of LC/ZnO composites is that lignin pellets are embedded in ZnO microplates. The lignin carbon in the composites mainly exists in an amorphous structure, and the specific surface area and pore channels of metal oxides are increased by the presence of lignin carbon. The electrochemical performance test shows that the carbonization temperature of LC/ZnO with the highest specific capacitance is 550 °C, and the capacitance retention rate reaches 96.74% after 1000 cycles of testing, indicating that the composite material has good cycle stability. Compared with the control group, it is found that the specific capacitance of LC/ZnO-550 °C is 2.3 times and 1.8 times that of ZnO-550 °C and LC-550 °C, respectively. This shows that during the electrochemical test, the lignin carbon and the metal oxide promote each other and act synergistically. In addition, the composite material exhibits the characteristics of a pseudo-capacitance capacitor, indicating that the redox reaction occurred in the electrochemical performance test.

## 1. Introduction

With the rapid development of the economy, the increasing level of industrialization, and the rapid growth of population, the existing resources developed by humankind have reached an unprecedented level, which has caused various problems such as waste of resources and environmental pollution. These problems threaten the natural environment that humankind depends on for survival and restricts the economic development of all countries in the world. Therefore, the development of clean, energy efficient, and renewable resources has become a hotspot of unanimous research by scientists, and biomass energy has received widespread attention as one of many renewable resources. Among them, lignin is favored by scholars for its advantages of large reserves, a wide range of sources, and environmental protection. As a largely agricultural country, China produces about 50 million tons of industrial lignin (it is the main by-product of papermaking and biorefinery industry) every year [1,2], but more than 95% of the industrial lignin is not recycled effectively; it is directly discharged into water bodies, causing environmental pollution and waste of resources [3]. Therefore, the high-value application of industrial lignin is of great significance to the recycling of resources, the protection of the environment, and the development of new materials. In addition to biomass energy, wind energy, solar energy, tidal energy, etc. are also well-known renewable energy sources, but the utilization of most of them will be affected by climate, environment, and seasons. High-efficiency energy storage devices are needed to deal with these green energy sources for storage and conversion [4]. Supercapacitors (also known as electrochemical capacitors), as a kind of energy storage devices with high power, fast charging and discharging performance, and long cycle life, are widely used in various aspects [5,6,7,8,9,10,11] such as hybrid electric vehicles, digital communication equipment, storage backup system, smart power grid, energy development, electronic products, etc.

As one of the electrode materials for supercapacitors, metal oxides will not be used as the electrode material for supercapacitors alone due to their disadvantages such as poor conductivity, short cycle life, and low-rate performance. However, we can use the advantages of porous carbon materials such as good conductivity to compound metal oxides to solve this problem. Therefore, researchers have found that using porous carbon materials to compound metal oxides can effectively improve the electrochemical properties of metal oxides [12]. In recent years, many carbon/metal oxide composites with good properties of supercapacitors have been widely reported, for example, graphene/cobalt tetroxide (GR/Co_3_O_4_) [13], porous carbon/manganese dioxide (PC/MnO_2_) [14], carbon nanotubes/zinc oxide (CNTs/ZnO) [15], etc. [16,17,18,19]. Lignin is a polymer compound with a three-dimensional network structure. Carbonization can form a graphene-like material with a certain pore structure (referred to as lignin carbon), which is an effective substitute for carbon-based materials. If lignin carbon is combined with metal oxides, it can improve the photoelectric properties of metal oxides and provide a favorable way for the resource utilization of industrial lignin. At present, most researchers use plant-based carbon as the electrode material for supercapacitors. For example, Xu et al. used wheat gluten as a carbon source to prepare a carbon material as a supercapacitor electrode material. According to the test, its specific capacitance can reach 350 F/g, indicating that the carbon material has excellent electrochemical properties [20]. Sevilla et al. used eucalyptus sawdust as a carbon source to prepare a supercapacitor carbon electrode material, which showed high specific capacitance in different electrolytes [21]. It can be seen that plant-based lignin carbon is a good carbon electrode material for supercapacitors [22,23,24]. In addition, industrial lignin can also be used as a carbon source to composite with metal oxides to prepare electrode materials for supercapacitors. For example, Li et al. prepared supercapacitor electrode materials by using activated carbon derived from walnut shell and zinc oxide, and the results showed that the specific capacitance of the composite material was 117.4 F/g, which basically remained stable after 1000 cycle tests [25]. Yun et al. used hardwood lignin as the carbon source to prepare carbon nanofibers and zinc oxide electrode materials with a specific capacitance of 165.0 F/g and capacitance retention of 94% after 3000 cycle tests [26]. Chen et al. used lignosulfonate as a carbon source and compounded it with metal oxide NiO to prepare NiO@MPC composite material and used it as a supercapacitor electrode material. Studies have shown that the material has a high specific capacitance (880.2 F/g), and the capacitance retention rate is 93.7% after 1000 charge and discharge cycles [27]. Therefore, the combination of lignin carbon and metal oxides can improve the electrochemical properties of single metal oxides. Researchers have tried different lignin carbon sources (such as lignosulfonate, walnut shell, and hardwood lignin), but the preparation of supercapacitor electrode materials using alkali lignin as carbon source and metal oxides is rarely reported. As ZnO has the advantages of wide temperature range, low cost, easy production, and single oxide form, we plan to use alkali lignin as the carbon source and combine it with ZnO to prepare electrode materials for supercapacitors in order to use the synergy between lignin carbon and metal oxides to improve the capacitance performance of metal oxides as electrode materials; it also provides an effective way for the high-value utilization of alkali lignin. At the same time, it also provides a theoretical basis for the wider application of lignin carbon/metal oxide composite electrode materials.

## 2. Results and Discussion

### 2.1. XRD Characterization Analysis

**Figure 1:**
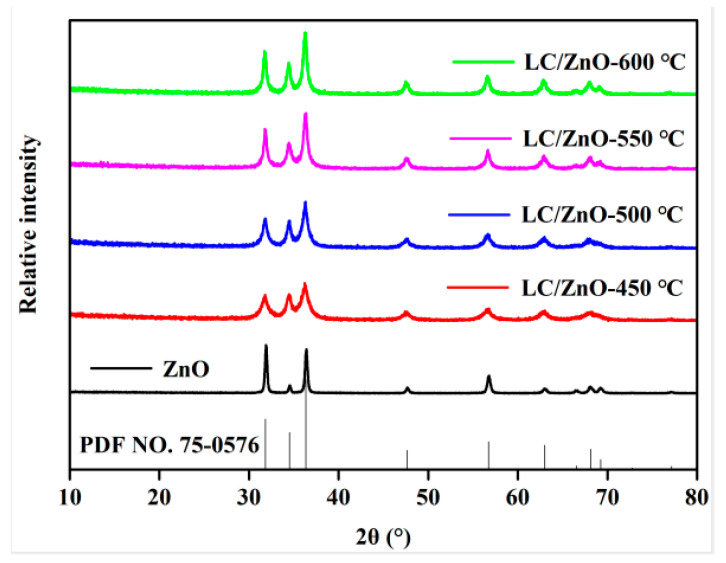
XRD patterns of LC/ZnO composites.

Figure 1 shows the XRD patterns of LC/ZnO composites at different calcination temperatures. As can be observed from Figure 1, the characteristic diffraction peaks of ZnO at 2θ of 31.7°, 34.4°, 36.4°, 47.5°, 56.7°, 62.9°, 66.4°, 67.9°, and 69.2° are consistent with those of the standard ZnO (PDF No. 36-1451) card, indicating that the crystal forms of both the prepared ZnO and the ZnO in the composite materials belong to hexagonal wurtzite [28,29]. In addition, no other XRD peaks are found in the XRD pattern of ZnO, indicating that there are no impurities in the prepared ZnO samples. With the gradual increase in the calcination temperature, the diffraction peaks of the metal oxide in the composite material become sharper, indicating that the increase in the calcination temperature is conducive to the formation of oxide crystals. In addition, with the addition of lignin carbon, although the XRD characteristic diffraction peaks of metal oxides in the composite material have changed, the main peak shape has not changed. It shows that LC/ZnO is successfully synthesized, and the addition of lignin carbon does not change the structure of the metal oxide. Moreover, the XRD pattern of the LC/ZnO composite material does not show an obvious diffraction peak of lignin carbon, which is because the lignin carbon mainly exists in the composite material in an amorphous structure. 

### 2.2. FESEM Characterization Analysis

**Figure 2:**
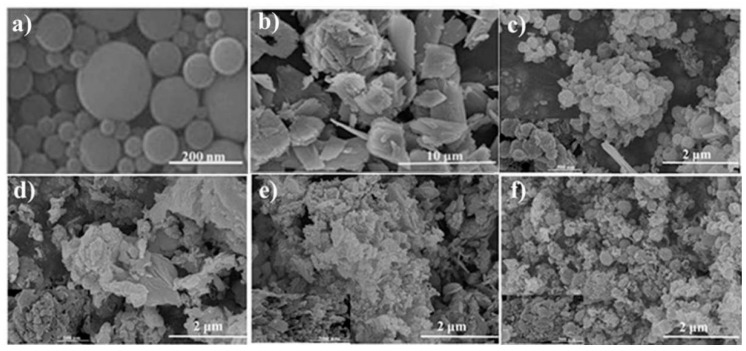
SEM images of LC/ZnO (**a**) QAL; (**b**) ZnO; (**c**) LC/ZnO-450 °C (inset: SEM image of 500 nm); (**d**) LC/ZnO-500 °C (inset: SEM image of 500 nm); (**e**) LC/ZnO-550 °C (inset: SEM image of 500 nm); (**f**) LC/ZnO-600 °C (inset: SEM image of 500 nm)).

Figure 2 shows the scanning electron micrographs of LC/ZnO at different calcination temperatures, QAL and ZnO. Figure 2a is the SEM image of QAL. It can be seen that QAL is composed of many nanospheres with smooth surfaces. It can be inferred from Figure 2b that ZnO alone is composed of a large number of irregular microplates with holes on the surface. Figure 2c–f is the scanning electron micrographs of LC/ZnO at different calcination temperatures. It can be observed that with the recombination of lignin, the morphology of ZnO microplates changes from rectangles with different lengths and widths to elliptical microplates. Additionally, with the increase in the calcination temperature, the ZnO microplates agglomerate. At 450 °C, the pores on the surface of the ZnO microplates in LC/ZnO disappear, and its surface becomes rough because the lignin nanospheres begin to be embedded in the ZnO microplates. Additionally, when the carbonization temperature continues to rise to 600 °C, the surface of the ZnO microplates in the LC/ZnO gradually changes from roughness to smoothness. These phenomena prove the successful synthesis of LC/ZnO.

### 2.3. FT-IR and Raman Characterization Analysis

**Figure 3:**
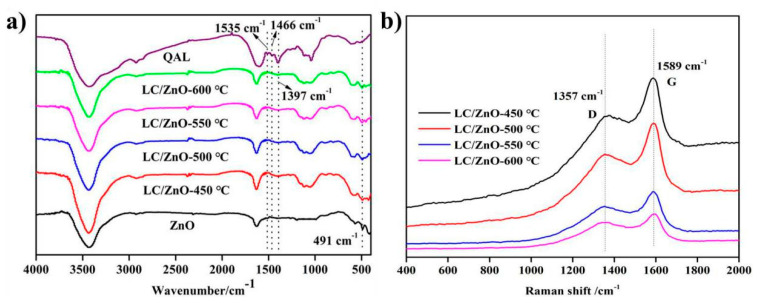
Spectra of LC/ZnO: (**a**) FT-IR; (**b**) Raman.

Figure 3 shows the FT-IR and Raman spectra of LC/ZnO at different calcination temperatures. Figure 3a shows the infrared spectra of LC/ZnO at different calcination temperatures. The peak at 1535 cm^−1^ is the stretching vibration of C=O in the aromatic ring [28], the peak at 1466 cm^−1^ corresponds to the stretching vibration of the C-OH bond in the aromatic ring [30], and the peak at 1397 cm^−1^ corresponds to the stretching vibration of the C-H bond in the alkyl group. Comparing the composite material with the uncarbonized QAL, it is found that the peaks of C=O, C-OH, C-H, etc. in the lignin disappear, indicating that the lignin is carbonized. In addition, the infrared spectrum of LC/ZnO is similar to that of ZnO alone. ZnO alone has a characteristic peak of Zn-O bond at 491 cm^−1^, and the peak of 491 cm^−1^ exits in the infrared spectrum of LC/ZnO, indicating that LC/ZnO has been successfully made. Moreover, with the increase in the calcination temperature of the composite material, the peak of 491 cm^−1^ in the infrared spectrum of LC/ZnO becomes more and more obvious, indicating that increasing the temperature is beneficial to the formation of Zn-O. Figure 3b is the Raman spectra of LC/ZnO at different calcination temperatures. It is known that the peak at 1357 cm^−1^ is the D peak, which indicates the presence of disorder and defects in the carbon structure. Additionally, the peak at 1589 cm^−1^ is the G peak, which reflects the presence of disordered sp^2^ hybrid carbon in the carbon structure. I_D_/I_G_ (intensity ratio) is generally used to estimate the degree of disorder in sp^2^ hybridized carbon regions [12]. The higher the I_D_/I_G_ is, the higher the degree of disorder. According to the Raman spectrum, the I_D_/I_G_ of LC/ZnO-450 °C, LC/ZnO-500, LC/ZnO-550 °C, and LC/ZnO-600 °C are 0.765, 0.769, 0.784, and 0.786, respectively. As the temperature increases, I_D_/I_G_ also increases, indicating that the sp^2^ hybrid carbon region becomes more disordered under higher temperature conditions, and more defects are formed, which is not conducive to the study of electrochemical performance. 

### 2.4. TG Characterization Analysis

**Figure 4:**
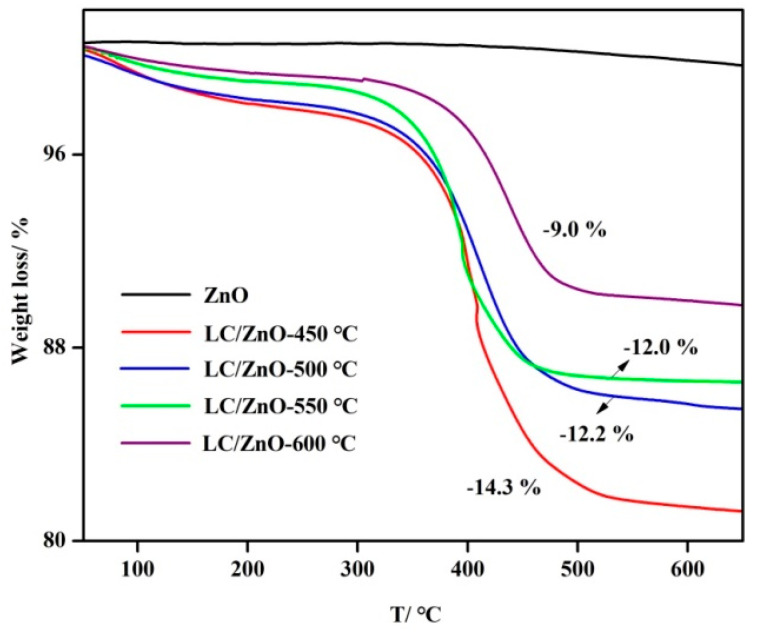
TG image of LC/ZnO composite.

Figure 4 shows the TG graphs of LC/ZnO at different calcination temperatures. It can be seen from the figure that lignin pyrolysis is basically divided into three stages. The first stage is the removal of free water below 200 °C. The second stage is the significant weight loss caused by the breakage of a large number of functional group branches connecting the phenylpropane structure at 200–500 °C. The third stage is the condensation polymerization and rearrangement of the benzene ring above 500 °C. The carbon content of LC/ZnO at different calcination temperatures is 14.3% (LC/ZnO-450 °C), 12.2% (LC/ZnO-500 °C), 12.0% (LC/ZnO-550 °C), 9.0% (LC/ZnO-600 °C), respectively. It can be observed that as the calcination temperature increases, the carbon content in the composite material gradually decreases. This is because lignin is easily decomposed at high temperatures. Therefore, the higher the calcination temperature is, the lower the content of the lignin.

### 2.5. Characterization and Analysis of N_2_ Adsorption-Desorption

**Figure 5:**
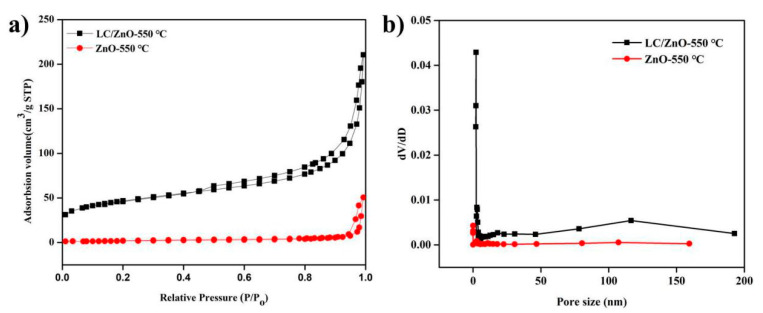
Nitrogen adsorption and desorption isotherms and pore size distribution of LC/ZnO-550 °C and ZnO-550 °C: (**a**) nitrogen adsorption and desorption isotherms; (**b**) pore size distribution.

According to the previous characterization analysis and the subsequent electrochemical performance research results, we measured the specific surface area and pore diameter of LC/ZnO-550 °C and ZnO-550 °C, respectively. It can be seen from Figure 5a that the adsorption isotherm of LC/ZnO-550 °C conforms to type IV isotherm, and the H_3_ hysteresis loop appears when P/P_0_ > 0.45, indicating that there are a lot of mesopores in the structure of LC/ZnO-550 °C. The N_2_ adsorption–desorption isotherm of ZnO-550 °C is a typical I-type isotherm at low pressure, and there is a hysteresis loop at P/P_0_ > 0.2, indicating that there are micropores in the ZnO structure. A typical IV-type isotherm is observed at medium pressure, and there is an H_3_ hysteresis loop at P/P_0_ > 0.75, indicating that mesopores exist in the ZnO structure. Secondly, under high-pressure conditions, LC/ZnO-550 °C has a larger adsorption capacity than ZnO-550 °C, indicating that the structure of LC/ZnO-550 °C contains more abundant pores [31]. According to Figure 5b, the pore structure type of ZnO-550 °C may be composed of micropores, mesopores, and macropores, while the pore structure type of LC/ZnO-550 °C may be composed of mesopores and macropores. The change in the structure of the hole can be proved from the scanning electron microscope (Figure 2b,e). In addition, the specific surface area (157.60 m^2^/g) of LC/ZnO-550 °C is 23.2 times that of ZnO-550 °C (6.79 m^2^/g). Additionally, according to the analysis of scanning electron microscope (Figure 2b,e), LC/ZnO-550 °C increases the specific surface area by expanding the pore volume, and this can also be achieved by LC/ZnO-550 °C (0.21 m^3^/g) and ZnO-550 °C (0.02 m^3^/g) pore volume size.

### 2.6. Cyclic Voltammetry

**Figure 6:**
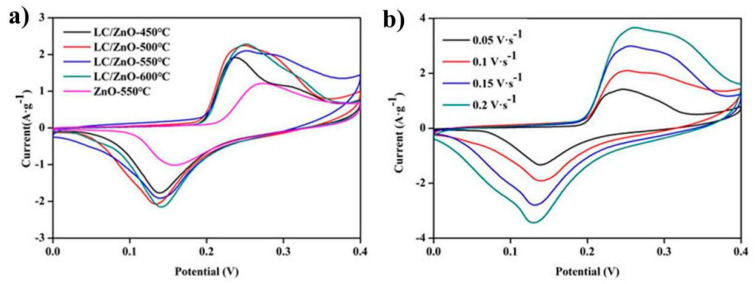
(**a**) CV curves of LC/ZnO at scan rates of 0.1 V·s^−1^; (**b**) CV curves of LC/ZnO-550 °C at different scan rates.

Figure 6a shows the CV curves of LC/ZnO at different calcination temperatures and ZnO alone in a three-electrode system at a sweep rate of 0.1 V/s. It can be seen from the figure that the CV curves of LC/ZnO all show obvious redox peaks. This feature is the characteristic of pseudo-capacitance capacitors, indicating that the capacitors formed by LC/ZnO are all pseudo-capacitance capacitors. The upwardly protruding peak is an oxidation peak, indicating that an oxidation reaction (oxygen evolution reaction) has occurred. The downwardly protruding peak is a reduction peak, indicating that a reduction reaction (hydrogen evolution reaction) has occurred. In addition, the area that is enclosed by the CV curve can be regarded as the specific capacitance of composite material. It can be seen from Figure 6a that the area enclosed by the CV curve of LC/ZnO-550 °C is the largest; therefore, it has the highest specific capacitance, and its specific capacitance is larger than that of ZnO-550 °C, indicating that the presence of lignin carbon greatly improves the electrochemical performance of metal oxides. Figure 6b shows the CV curve of LC/ZnO-550 °C tested at different scanning speeds. It can be observed from Figure 6b that the larger the scanning speed is, the larger the area covered by the CV curve, indicating that increasing the scanning speed can increase the specific capacitance of the composite material. Additionally, when the scanning speed is increased to 0.2 V/s, the curve still maintains the original redox peak, indicating that LC/ZnO-550 °C has a good rate performance. 

### 2.7. Galvanostatic Charge/Discharge

**Figure 7:**
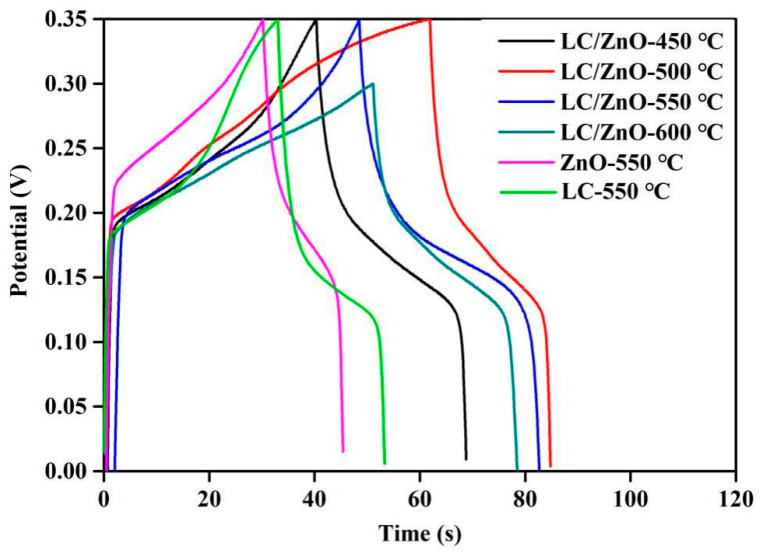
GCD curves at 0.1 A/g of the LC/ZnO composites.

Figure 7 shows the GCD curves of LC/ZnO at different calcination temperatures, ZnO and LC at 0.1 A/g. It can be seen that the GCD curve of LC/ZnO at each temperature shows a clear charge and discharge platform, indicating that a significant oxidation-reduction reaction occurred during the charge and discharge process. This result is consistent with the analysis result of cyclic voltammetry. According to Formula (5), the weight-specific capacitances of LC/ZnO-450 °C, LC/ZnO-500 °C, LC/ZnO-550 °C, and LC/ZnO-600 °C are calculated as 65 F/kg, 81 F/kg, 101 F/kg, and 92 F/kg, respectively. It can be inferred that the weight-specific capacitance of the composite material shows a trend of first increasing and then decreasing as the temperature rises, and the weight-specific capacitance of LC/ZnO reaches the highest at 550 °C. According to Formulas (6) and (7), the energy density and power density of the composite materials are shown in Table 1. The energy densities of LC/ZnO-450 °C, LC/ZnO-500 °C, LC/ZnO-550 °C, and LC/ZnO-600 °C are calculated as 3.98 W·s/kg, 4.96 W·s/kg, 6.19 W·s/kg, and 5.64 W·s/kg, and the power density is 0.173 W/kg, 0.177 W/kg, 0.177 W/kg and 0.176 W/kg, respectively. In addition, the weight-specific capacitance of LC/ZnO-550 °C is 2.3 times that of ZnO-550 °C (43 F/kg) and 1.8 times that of LC-550 °C (57 F/kg). It can be inferred that the combination of lignin carbon and metal oxide can effectively improve the electrochemical performance of each other. This is because they both synergize and promote each other. The metal oxide protects the lignin from being decomposed, and lignin increases the specific surface area and pores of metal oxides.

### 2.8. Electrochemical Impedance Spectroscopy

**Figure 8:**
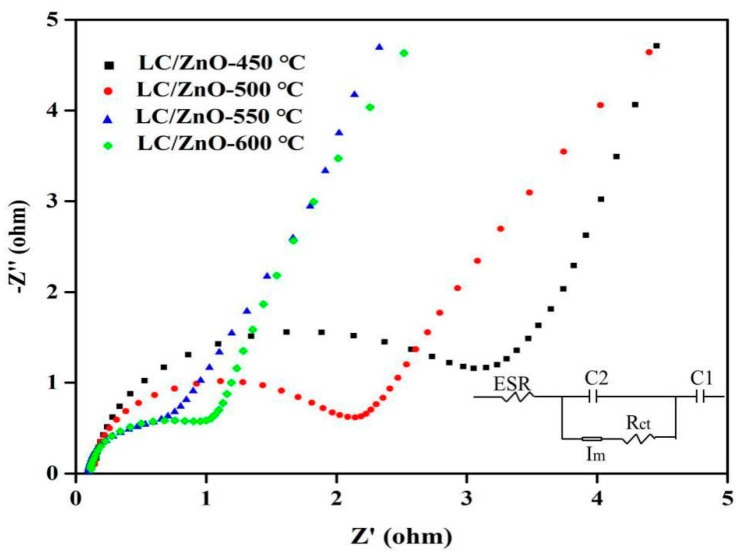
Nyquist plot and fitted equivalent circuit diagrams (inset) of LC/ZnO composites at different temperatures (C1 refers to the external power supply. C2 refers to the three-electrode system. Rct refers to the charge transfer resistance of the interface between the LC/ZnO electrode material and the electrolyte. ESR refers to the the equivalent series resistance of the composite material. Im is equivalent to a galvanometer).

Figure 8 shows the electrochemical impedance spectroscopy of LC/ZnO at different calcination temperatures. It can be observed from Figure 8 that the LC/ZnO at different calcination temperatures all appear to be semicircular in the high-frequency region, and there is a nearly 90° straight line in the low-frequency region, indicating that LC/ZnO with different calcination temperature all show good capacitance performance. In the high-frequency region, the charge transfer resistance (Rct) of the interface between the LC/ZnO electrode material and the electrolyte can be estimated based on the diameter of the semicircle in the Nyquist diagram. This resistance comes from the electron transfer process on the electrode surface [30]. According to the fitted equivalent circuit, the charge transfer resistance contained in the LC/ZnO at each calcination temperature is about 1.28 Ω (LC/ZnO-450 °C), 1.10 Ω (LC/ZnO-500 °C), 0.47 Ω (LC/ZnO-550 °C), and 0.58 Ω (LC/ZnO-600 °C). At the same time, the steepness of the linear curve in the low-frequency region indicates the size of the diffusion resistance. The steeper the curve is, the smaller the diffusion resistance, the flatter the curve, and the greater the diffusion resistance. Therefore, LC/ZnO with different calcination temperatures basically exhibits a typical capacitance behavior with low diffusion resistance [32]. In addition, in the high-frequency region, the intercept of the Nernst curve and the abscissa represents the equivalent series resistance (ESR) of the composite material. ESR is composed of the resistance of the electrolyte and the contact resistance and the inherent resistance of the composite material and the nickel foam [30]. The equivalent series resistance of LC/ZnO in supercapacitors are 0.16 Ω (LC/ZnO-450 °C), 0.13 Ω (LC/ZnO-500 °C), 0.09 Ω (LC/ZnO-500 °C), and 0.11 Ω (LC/ZnO-600 °C), respectively. It can be seen that the resistance of LC/ZnO supercapacitors at different temperatures shows a trend that first decreases and then rises as the temperature rises, and the resistance of LC/ZnO supercapacitors reaches the lowest at 550 °C. In summary, the supercapacitor based on LC/ZnO-550 °C has the lowest resistance; therefore, it is considered that it has the best electrochemical performance.

### 2.9. Cycle Performance Test

**Figure 9:**
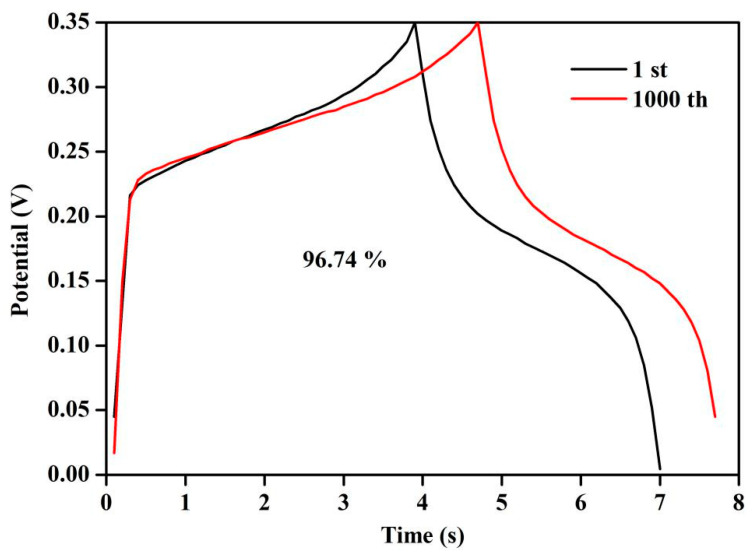
GCD curves at 1st and 1000th cycles of LC/ZnO-550 °C composite.

In this thesis, the GCD of the LC/ZnO-550 °C was carried out several times under the current density of 1 A/g to study its cycle performance. Figure 9 shows the GCD curve of LC/ZnO-550 °C at the 1st and 1000 cycles. It can be seen that after 1000 times of charging and discharging, the capacitance retention rate reached 96.74%. In addition, after 1000 cycles, the GCD curve of LC/ZnO-550 °C maintains the same shape as the first time, indicating that LC/ZnO-550 °C has good cycle stability. 

### 2.10. Mechanism Analysis of Electrochemical Performance

In the three-electrode system, when there is no external power supply, the positive and negative ions in the KOH solution present a disordered state. When there is an external power source, electrons flow from the positive electrode to the negative electrode (composite material). The K^+^ moves to the negative electrode, and OH^-^ msoves to the positive electrode due to the electrostatic effect in the KOH solution. As the composite material has a large specific surface area and abundant pores, electrons can be accumulated on the surface and the pores, and hydrogen evolution occurs. The hydrogen evolution chemical reaction equation of LC/ZnO is shown in the following Formula (2). The oxygen evolution reaction occurs on the surface of the positive electrode, and the chemical reaction equation is shown in the following Formula (1). In this case, a large amount of K^+^ in the KOH solution moves to the negative electrode, and a large amount of OH^-^ moves to the positive electrode; hence, energy is stored. This process is a charging process. The discharging process is the reverse process of the charging process. In this process, the composite material itself acts as a negative electrode, and it will lose the electrons accumulated on its surface and in the pores during the charging process, and an oxygen evolution reaction will occur. The oxidation reaction of LC/ZnO is shown in the following Formula (3). At this time, the OH^-^ in the KOH solution moves to the LC/ZnO electrode material. The platinum wire electrode is used as the positive electrode, the electrons losing the LC/ZnO electrode material will be obtained and the hydrogen evolution reaction will occur, as shown in the following Formula (4). Additionally, the K^+^ in the KOH solution moves to the platinum wire electrode; hence, energy is released.

The charging process of LC/ZnO is as follows:

(1) $$4OH^{-}\to O_{2}+4e^{-}+2H_{2}O$$

(2) $$4H_{2}O+4e^{-}\to 2H_{2}+4OH^{-}$$

The discharge process of LC/ZnO is as follows:

(3) $$4OH^{-}\to O_{2}+4e^{-}+2H_{2}O$$

(4) $$2H_{2}O+2e^{-}\to H_{2}+2OH^{-}$$

## 3. Materials and Methods

### 3.1. Preparation Method of Composite Material

#### 3.1.1. Activation of Alkali Lignin

**Figure 10:**
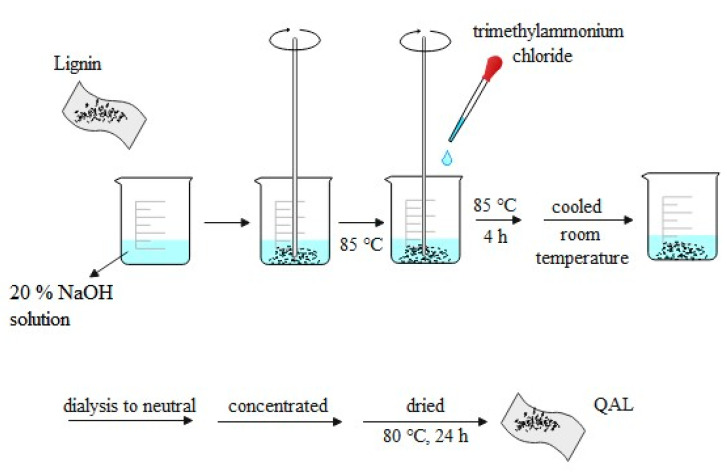
Activation of alkali lignin.

As shown in Figure 10, 9.02 g of alkali lignin was dissolved in 20% NaOH solution, stirred for 30 min, and heated to 85 °C; then, 7.51 g of trimethylammonium chloride was added drop by drop, kept at 85 °C for 4 h, and then cooled to room temperature. The cooling product was transferred to a dialysis bag for dialysis to neutral. After that, the dialysis product was concentrated at 80 °C to viscous and dried at 80 °C for 24 h to obtain the activated product of alkali lignin, which was denoted as QAL.

#### 3.1.2. Preparation of Lignin Carbon/Zinc Oxide Composite Material

**Figure 11:**
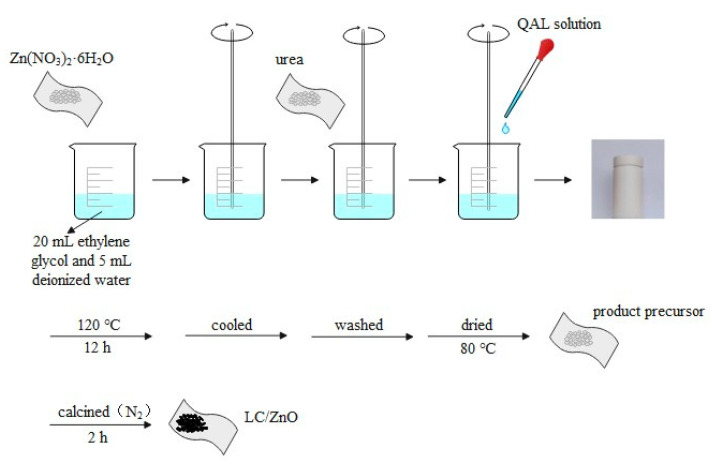
Preparation of LC/ZnO composite material.

As shown in Figure 11, 0.297 g Zn(NO_3_)_2_·6H_2_O was dissolved in 20 mL ethylene glycol and 5 mL deionized water and stirred for 30 min; then, 0.6 g urea was added and continued stirring for 30 min, after which QAL solution (take 0.1 g QAL dissolved in 10 mL deionized water) was added drop by drop, stirred for another 30 min, and then the reaction system was transferred to a polytetrafluoroethylene stainless steel reactor, reacted at 120 °C for 12 h, and cooled to room temperature. It was washed 3 times with deionized water and absolute ethanol and dried at 80 °C for 12 h to obtain the product precursor. The product precursor was calcined at a certain temperature (N_2_ atmosphere) to obtain lignin carbon/zinc oxide, which is recorded as LC/ZnO.

### 3.2. Characterization of Composite Materials

X-ray powder diffraction (XRD) was used to test the crystalline form of the composite material. The surface morphology of the composite material was observed by field emission scanning electron microscopy (FESEM). The group and carbon structure of composite materials was tested through Fourier transform infrared spectroscopy (FT-IR) and Raman Spectroscopy (Raman). The carbon content of composite materials was determined by thermogravimetry (TG, temperature range 25–700 °C, heating rate: 20 °C/min, protective gas: N_2_ (99.99%)). The specific surface area and pore structure of composite materials was determined through N_2_ adsorption–desorption.

### 3.3. Electrochemical Performance Test

#### 3.3.1. Production of Electrode Materials

The composite material (LC/ZnO, 0.08 g), polytetrafluoroethylene (as adhesive), and conductive carbon black (as conductive agent) with a mass ratio of 80:10:10 were dispersed into 50 mL anhydrous ethanol, stirred for 30 min, and heated at 80 °C for 30 min; the elastic solid shaped like plastic clay was rolled onto the foamed nickel and then dried at 80 °C for 12 h; it was used as the working electrode for standby.

#### 3.3.2. Cyclic Voltammetry Curve Test

The saturated calomel electrode was used as the reference electrode, and the platinum electrode was used as the counter electrode. A three-electrode system was formed with the working electrode, the reference electrode, and the counter electrode. The electrochemical performance was tested in a 6 mol/L sodium hydroxide solution at room temperature.

Cyclic voltammetry (CV) was used to test the cyclic voltammetry curve of the composite material. The scanning speed of LC/ZnO was 0.05 V/s, 0.1 V/s, 0.15 V/s, and 0.2 V/s, and the voltage range was 0~0.4 V.

#### 3.3.3. Determination of Specific Capacitance

The composite material was tested by the galvanostatic charge/discharge (GCD) method at a current density of 0.1 A/g, and the voltage range of LC/ZnO was 0~0.35 V. The following formulas are used to calculate the weight-specific capacitance (*C*, F/kg), energy density (*E*, W·s/kg), and power density (*P*, W/kg) of the composite material.

(5) $$C=\frac{I\Delta t}{m\Delta V}$$

(6) $$E=\frac{CV^{2}}{2}$$

(7) $$P=\frac{E}{\Delta t}$$

where *I* is the discharge current; Δ*t* is the discharge time; *m* is the mass of the active material; Δ*V* is the potential drop during the discharge.

#### 3.3.4. Simulation of Equivalent Circuit and Determination of Dynamic Parameters

The equivalent circuit of the electrode system is inferred through the electrochemical impedance spectroscopy (EIS) test, the action mechanism of the composite material in the electrode system is studied, and the kinetic parameters are estimated. The testing method consisted of determining the value of the open-circuit voltage of each composite material and then using the open-circuit voltage to perform an AC impedance test on the composite material.

## 4. Conclusions

In this paper, ZnO and lignin carbon are used to prepare composite materials (LC/ZnO), and their characterization and electrochemical performance test are carried out. The results show that the LC/ZnO composite material is lignin pellets embedded in ZnO microplates. The lignin carbon in the composite mainly exists in an amorphous structure, and the presence of lignin carbon increases the specific surface area and pores of the metal oxide. The composite material undergoes an oxidation-reduction reaction in the electrochemical performance test. LC/ZnO has the highest weight-specific capacitance at 450 °C, and the specific capacitance of LC/ZnO-550 °C is 2.3 times and 1.8 times that of ZnO-550 °C and LC-550 °C, respectively. It can be inferred that lignin carbon and metal oxide synergize and promote each other during the recombination process. At the same time, after 1000 cycles test of LC/ZnO-550 °C, the capacitance retention rate reached 96.74%. It shows that the composite material (LC/ZnO-550 °C) has the characteristics of a pseudocapacitance capacitor and good cycle stability.

## Figures and Tables

**Table 1:** Energy density and power density of LC/ZnO composites.

Composites	LC/ZnO-450 °C	LC/ZnO-500 °C	LC/ZnO-550 °C	LC/ZnO-600 °C
Energy density(W·s/kg)	3.98	4.96	6.19	5.64
Power density(W/kg)	0.173	0.177	0.177	0.176

## Data Availability

The data presented in this study are available within the article.

## Sample Availability

Sample of the compounds are available from the authors.
